# Supplementary material for: Longitudinal Associations Between Depressive Symptoms and Brain Structure Across Late Childhood and Adolescence: A Panel Network Analysis Study
Source: Biol Psychiatry Glob Open Sci. 2026 Sep 9;7(1):100824. doi: 10.1016/j.bpsgos.2026.100824 (PMC13691503; doi:10.1016/j.bpsgos.2026.100824)
Supplement: Supplementary file 1 [file mmc1.pdf]

SUPPLEMENTARY INFORMATION

Longitudinal Associations Between Depressive Symptoms and Brain Structure Across Late Childhood and Adolescence: A Panel Network Analysis Study

Aksnes *et al.*

Table S1. Race/Ethnicity distribution at baseline

Race/Ethnicity	N	Percentage
Asian	213	2.3
Multiracial	890	9.5
Native American / Alaska Native	312	3.3
Non-Hispanic Black	1,478	15.8
Non-Hispanic White	5,887	62.7
Other / Missing	602	6.4

Note. Race/ethnicity categories are based on parent report. Percentages reflect the proportion of the full baseline sample (N = 9,556). Categories are listed in alphabetical order per BP reporting guidelines.

Table S2. Sample characteristics by timepoint and sex

TP	Male N	Female N	% Male	Age M (SD)	Age range
1	5,011	4,545	52.4%	9.94 (0.62)	8.32–11.30
2	3,543	3,015	54.0%	12.00 (0.65)	10.23–13.85
3	2,772	2,381	53.8%	14.14 (0.71)	12.51–16.58
4	3,254	2,913	52.8%	16.05 (0.67)	14.70–18.00

Note. TP = timepoint. Timepoint 1 represents baseline and timepoints 2–4 represent 2-year, 4-year, and 6-year follow-up assessments. N reflects the number of participants with available structural MRI and CBCL data at each timepoint. Sex % reflects the proportion of males and females within each timepoint. Age is reported in years.

Table S3. Depressive symptoms by timepoint and sex

Timepoint	Sex	Anhedonia	Worthlessness	Lethargy	Depressed Mood	Sleep Problems
TP1	Male	0.12 (0.36)	0.15 (0.39)	0.08 (0.30)	0.14 (0.38)	0.17 (0.43)
TP1	Female	0.07 (0.28)	0.11 (0.34)	0.07 (0.29)	0.12 (0.34)	0.15 (0.40)
TP2	Male	0.15 (0.41)	0.15 (0.39)	0.14 (0.39)	0.15 (0.39)	0.21 (0.48)
TP2	Female	0.12 (0.36)	0.14 (0.38)	0.13 (0.37)	0.18 (0.42)	0.22 (0.48)
TP3	Male	0.19 (0.44)	0.14 (0.37)	0.16 (0.41)	0.17 (0.41)	0.22 (0.48)
TP3	Female	0.18 (0.43)	0.17 (0.42)	0.19 (0.44)	0.29 (0.52)	0.28 (0.53)
TP4	Male	0.17 (0.43)	0.13 (0.37)	0.14 (0.39)	0.17 (0.42)	0.21 (0.46)
TP4	Female	0.17 (0.42)	0.17 (0.42)	0.19 (0.44)	0.26 (0.50)	0.27 (0.53)

Note. CBCL parent-reported depressive symptom items scored 0–2; higher scores indicate greater symptom severity. Mean (SD). TP = timepoint.

Table S4. Brain measures by timepoint and sex

Timepoint	Sex	Fusiform	Insula	mOFC	Cingulate	Hippocampus	Global SA
TP1	Male	2.88 (0.10)	3.16 (0.13)	2.65 (0.11)	2.68 (0.10)	−0.08 (1.01)	197,126 (16,400)
TP1	Female	2.89 (0.10)	3.15 (0.13)	2.65 (0.11)	2.69 (0.10)	−0.09 (0.94)	181,026 (15,089)
TP2	Male	2.85 (0.10)	3.13 (0.12)	2.61 (0.11)	2.65 (0.10)	−0.01 (1.01)	198,055 (16,405)
TP2	Female	2.85 (0.10)	3.10 (0.12)	2.60 (0.11)	2.65 (0.10)	−0.02 (0.95)	181,249 (15,086)
TP3	Male	2.82 (0.10)	3.10 (0.13)	2.56 (0.11)	2.61 (0.09)	0.05 (1.04)	197,156 (16,608)
TP3	Female	2.80 (0.10)	3.06 (0.12)	2.54 (0.10)	2.60 (0.10)	0.04 (0.97)	177,893 (15,042)
TP4	Male	2.80 (0.10)	3.08 (0.11)	2.53 (0.10)	2.58 (0.10)	0.09 (1.05)	195,010 (16,669)
TP4	Female	2.77 (0.10)	3.03 (0.12)	2.50 (0.10)	2.56 (0.10)	0.13 (1.03)	175,799 (15,156)

Note. Cortical thickness measures (Fusiform, Insula, mOFC, Cingulate) in mm, post-LongComBat harmonized. Hippocampus = ICV-adjusted residuals (z-scores). Global SA = global surface area in mm^2. Mean (SD). mOFC = medial orbitofrontal cortex. TP = timepoint.

ABCD Inclusion/Exclusion criteria

The ABCD exclusion criteria includes not being fluent in English language for the child (as cognitive testing and other materials were in English); severe sensory, intellectual, medical or neurological issues that would impact the child's ability to comfortably and validly complete the protocol and contraindications to MRI scanning. Parent/guardian exclusionary criteria were non fluency either English or Spanish. Additionally, participants with a current diagnosis of schizophrenia or autism spectrum disorder (moderate, severe) were also excluded (a past diagnosis that has remitted is not exclusionary, see 7.0 release notes).

MRI Acquisition and Processing

Structural T1-weighted MRI data were acquired as part of the Adolescent Brain Cognitive Development (ABCD) Study across multiple 3T scanner platforms (Siemens Prisma, GE 750, and Philips systems). Structural MRI data were processed centrally by the ABCD Study team using FreeSurfer (version 7.1.1; Fischl et al., 2002). Processing steps included motion correction and averaging, skull stripping, Talairach transformation, segmentation of subcortical white and deep gray matter structures, intensity normalization, tessellation of the gray–white matter boundary, automated topology correction, and cortical surface reconstruction (Casey et al., 2018; Hagler et al., 2019). Only scans passing ABCD structural quality control were retained. All ABCD Study data are stored in the National Institute of Mental Health Data Archive (NDA; Collection #2573) and accessed by registered and authorized users (Request #7474, principal investigator: LTW). The 7.0 data release has been assigned the DOI https://doi.org/10.82525/8f3w-5260. From the ABCD data repository (release 7.0), we extracted tabulated regional measures of cortical thickness and subcortical volume. For the present study, analyses focused on cortical thickness in the cingulate cortex, insula, medial orbitofrontal cortex, and fusiform gyrus, as well as hippocampal volume and surface area all derived from the Desikan-Killiany atlas (Desikan et al., 2006). Cortical thickness is defined as the outer layer of grey matter of the cerebral cortex and measured using T1-weigthed images to create a model of the white and pial surfaces before calculating thickness as the shortest vertex wise distance between these (Fischl & Dale, 2000). The calculation of surface area includes defining the boundaries of the grey and white matter, before summing the triangle areas converging at each particular vertex, resulting in global surface area (Norbom et al., 2021).

Structural MRI data were restricted to the four longitudinal sessions used in the main analyses (TP1–TP4: ses-00A, ses-02A, ses-04A, ses-06A). Within each wave, only observations passing T1-weighted structural quality control were retained. Wave-specific cortical thickness, surface area, and subcortical tables were merged by participant and session (with age and sex included as additional join keys to prevent duplication or misalignment) and stacked into a single long-format dataset. Before enforcing family independence, the pooled dataset comprised 33,456 observations from 11,794 unique participants. Wave-specific sample sizes were 11,554 (TP1), 7,995 (TP2), 6,315 (TP3), and 7,592 (TP4). To address relatedness within the ABCD cohort, one participant per family was retained across all waves. Participants were randomly selected using a fixed random seed to ensure reproducibility

(set.seed = 123). This global family filter reduced the dataset to 27,434 observations from 9,761 unique participants, corresponding to 2,031 individuals removed due to sibling or twin clustering. After adding scanner batch information and constructing the longCombat input dataset, all rows with complete core harmonization covariates (participant ID, timepoint, scanner batch, sex, age) were retained (0 missing across these variables). No listwise deletion was applied to MRI features, preserving natural longitudinal missingness across waves. For harmonization, the final longCombat-ready feature sets comprised 70 cortical thickness measures, 70 cortical surface area measures, and 42 subcortical (ASEG) volume measures.

Figure S1 – Number of timepoints contributed by sex.

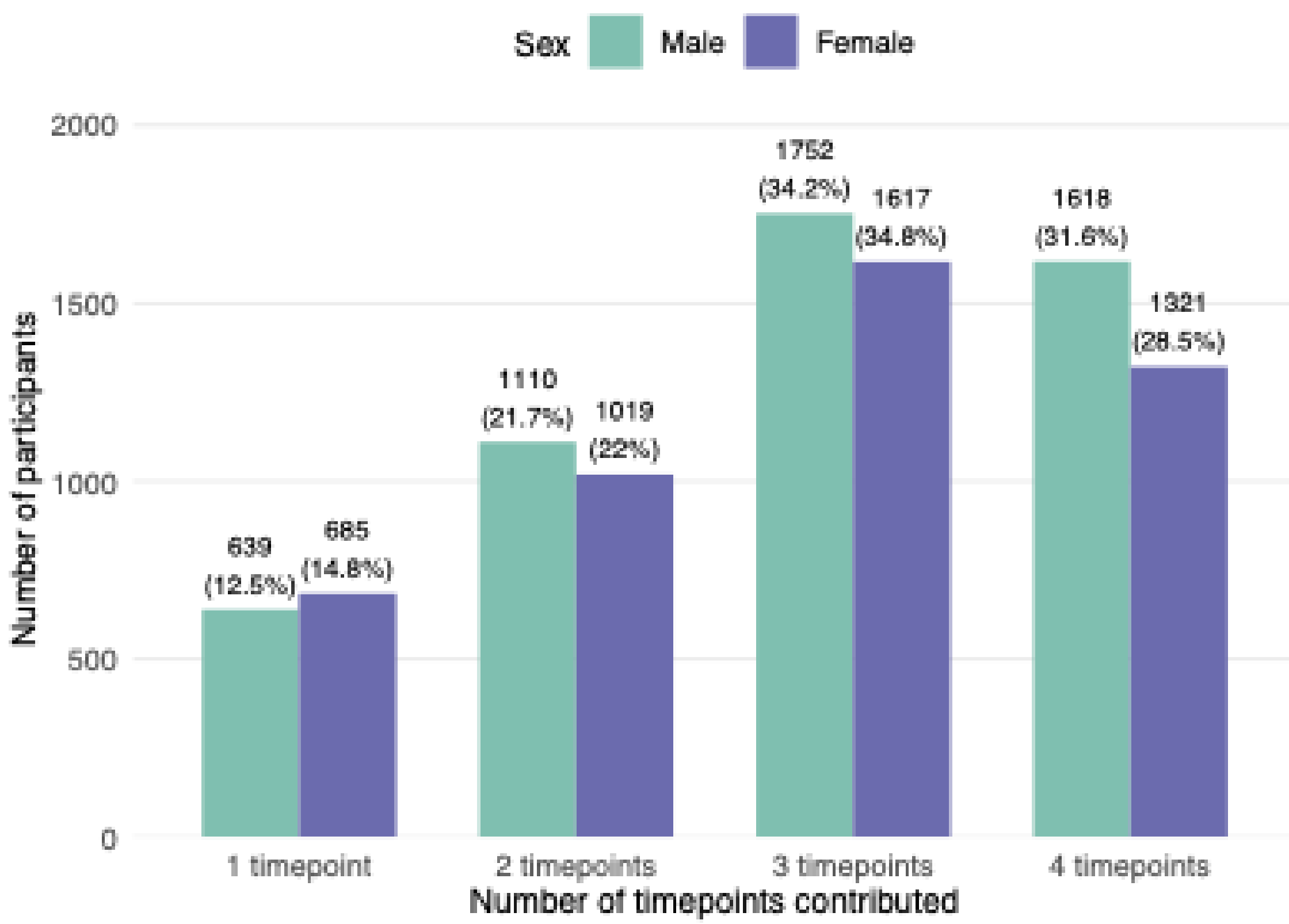

Note. Bars show the number of male and female participants contributing data at one, two, three, or four timepoints. Data availability was defined as having both CBCL symptom and structural brain data non-missing at a given timepoint. Percentages reflect the proportion within each sex.

longCombat Harmonization

Given the multi-site and longitudinal nature of the ABCD Study, structural MRI measures are susceptible to scanner- and site-related batch effects that may introduce non-biological

variability. To mitigate such effects while preserving true within-person change and between-person differences, we harmonized T1-derived structural MRI measures using longCombat (Beer et al., 2020). LongCombat extends the ComBat framework to longitudinal data by incorporating a mixed-effects model that accounts for repeated measures within individuals, estimating additive and multiplicative scanner effects while preserving subject-specific trajectories (Fortin et al., 2018). Harmonization was applied to data from baseline (ses-00A), 2-year (ses-02A), 4-year (ses-04A), and 6-year follow-up (ses-06A), after restricting the sample to participants whose scans passed ABCD structural quality control and after removal of related individuals. Scanner identity was derived from the ABCD administration tables and specified as a batch variable. Harmonization was conducted separately for cortical thickness (Desikan-Killiany atlas), subcortical volumes (aseg.stats) and global surface area, using the same covariate specification across modalities: age, sex, and timepoint, with a random intercept for participant ID. Following harmonization, bilateral averages were computed for each region of interest. Hippocampal volume was subsequently residualized for intracranial volume and sex.

Figure S2. Hippocampal volume before and after longCombat harmonization by scanner
batch

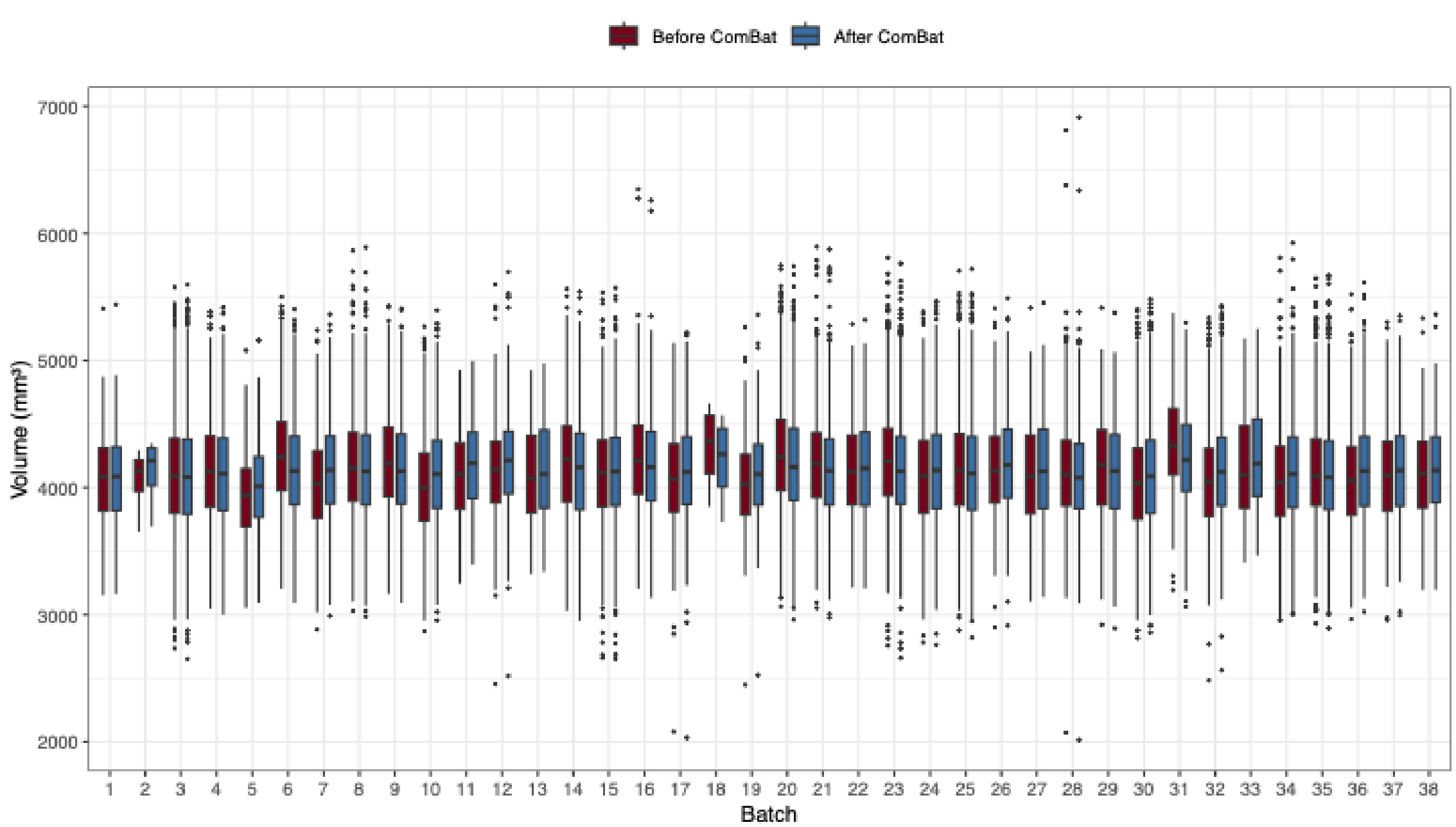

Figure S3. Insula cortical thickness before and after longCombat harmonization by scanner
batch

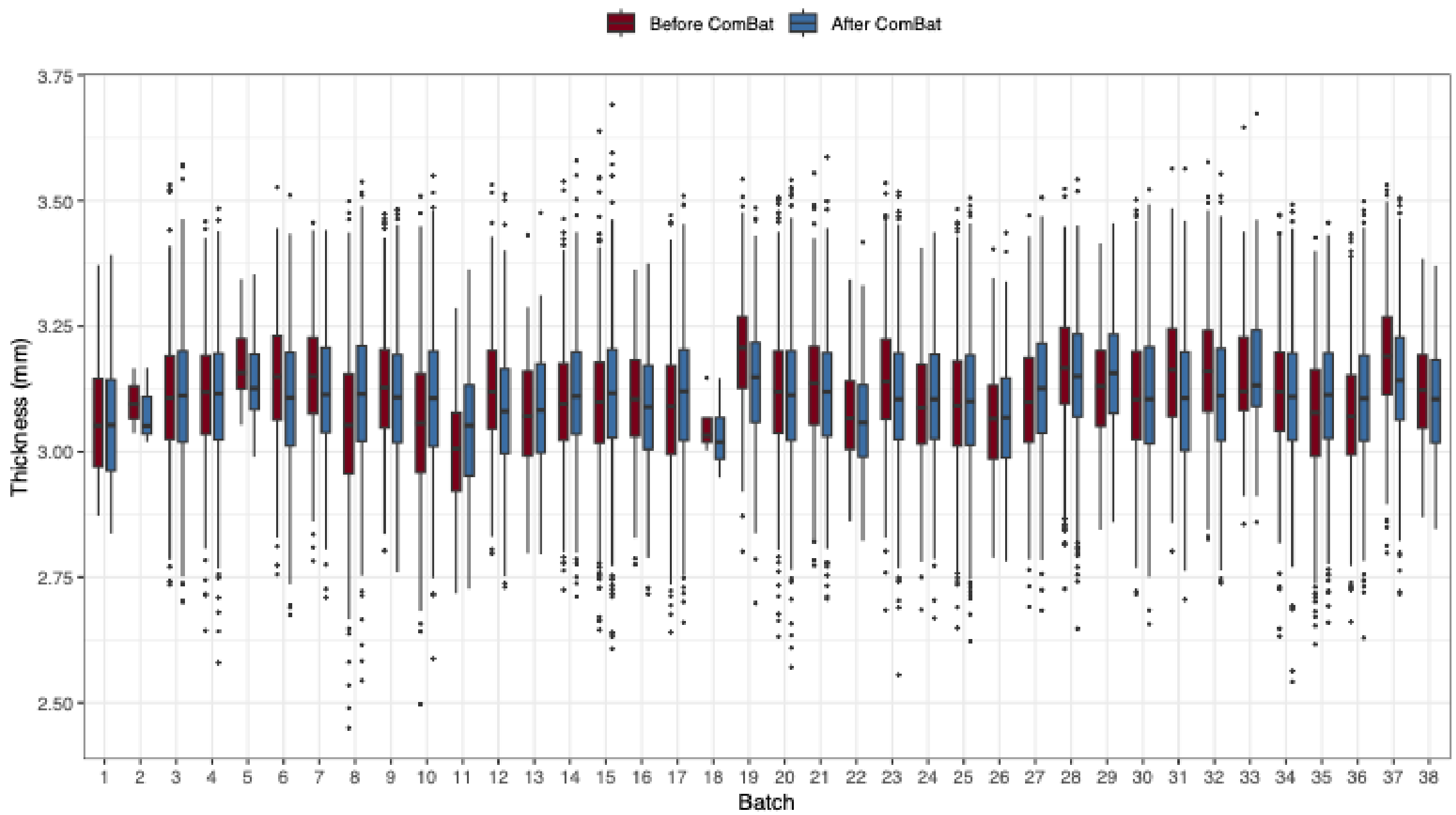

Note. Boxplots show the distribution of raw (before longCombat, red) and harmonized (after longCombat, blue) values for hippocampal volume and insula cortical thickness as representative examples, across scanner batches (N = 31 scanners). LongComBat harmonization was applied separately for cortical thickness, surface area, and subcortical volume using longitudinal mixed-effects models with age, sex, and timepoint as fixed-effects covariates and subject-level random intercepts to account for the repeated-measures structure of the data. Harmonization was performed across all regions of interest; figures for remaining regions are available upon request.

Time trend inspection and detrending

To evaluate the stationarity assumption underlying panel-GVAR models, we inspected mean trajectories of depressive symptoms and brain structural measures across the four timepoints prior to detrending. For each variable, we computed timepoint-specific means and 95% confidence intervals separately by sex (Supplementary Figure S4). Prior to model estimation, all variables were inspected for systematic time trends by fitting linear and quadratic mixed-effects models with participant-specific random intercepts, including Sex and TP × Sex interactions, and comparing model fit using BIC. Cubic trends were not considered given that the study comprises four timepoints, at which a cubic polynomial would have as many parameters as measurement occasions and risk overfitting the developmental trajectory; the

model with the lowest BIC was selected, with linear preferred in cases of equivalent fit. BIC comparisons indicated systematic mean-level change over time in several variables. Because such structured developmental trends violate the stationarity assumption of GVAR models and can induce spurious temporal associations, each variable was detrended by regressing it on the selected polynomial terms using ordinary least squares, and the residuals were retained for network estimation. This procedure removes systematic group-level developmental trends while preserving within-person deviations around individual means and stable between-person differences. Table S5 reports the selected trend order and BIC values for each variable, allowing readers to verify which trends were removed for each node.

Table S5. Detrending Model Selection

Available as separate Excel file: TableS5_detrending_model_selection

Figure S4. Mean trajectories over time

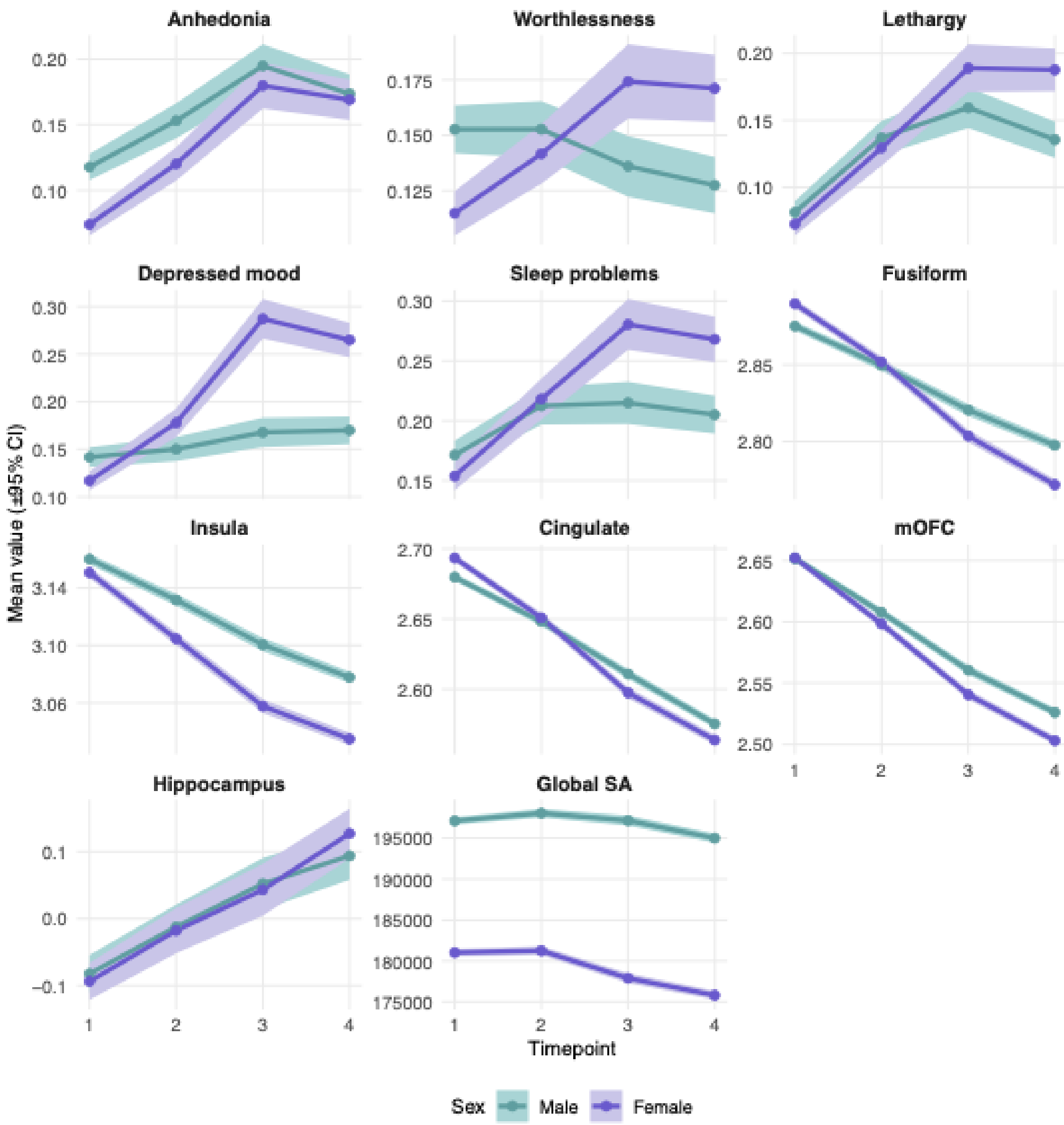

Note. Mean trajectories (±95% CI) across timepoints 1–4 for five depressive symptom items (anhedonia, worthlessness, lethargy, depressed mood, sleep problems) and six structural brain metrics (fusiform, insula, cingulate, mOFC, hippocampus, global surface area), shown separately for males (cadetblue) and females (purple). Timepoint 1 represents baseline and timepoints 2–4 represent 2-year, 4-year, and 6-year follow-up assessments. Trajectories are shown prior to detrending. Confidence intervals are plotted identically across all panels; differences in band visibility reflect genuine differences in between-person variability and measurement scale across variables rather than plotting errors.

Network invariance testing procedure

To examine sex differences in network structure, we conducted multigroup panel GVAR invariance testing using the *psychonetrics* package (Epskamp, 2025). A heterogeneous multigroup model was estimated in which all parameters of the temporal, contemporaneous, and between-person networks were freely estimated separately for males and females, and compared against a homogeneous model constraining corresponding parameters to be equal across sex. Model fit was evaluated using the Akaike Information Criterion (AIC), following recommendations by Hoekstra et al., (2024) for invariance testing in network models, with lower values indicating better model fit accounting for parsimony. This approach follows standard SEM-based procedures for testing structural invariance in longitudinal network models. Fit indices for all invariance models are reported in Tables S6.

Table S6. Fit Indices for Invariance Models

Comparison	Model	df	CFI	TLI	RMSEA	RMSEA 95% CI	AIC	BIC	$\Delta\chi^2$	Δdf	p
All networks	Different	1,540	.864	.831	.029	[.028, .030]	664,939	668,734	—	—	—
	Equal	1,771	.858	.840	.028	[.027, .028]	665,049	667,184	571.85	231	< .001
Within-person	Different	1,540	.864	.831	.029	[.028, .030]	664,939	668,734	—	—	—
	Equal (within)	1,716	.856	.837	.028	[.027, .029]	665,090	667,620	502.93	176	< .001
Between-person	Different	1,540	.864	.831	.029	[.028, .030]	664,939	668,734	—	—	—
	Equal (between)	1,595	.863	.832	.029	[.028, .029]	664,895	668,294	65.19	55	.163

Note. The "Different" model (configural) freely estimates all network parameters separately for males and females. The "Equal" model constrains all parameters to be equal across sex. "Equal (within)" constrains only within-person parameters; "Equal (between)" constrains only between-person parameters. df = degrees

of freedom; CFI = Comparative Fit Index; TLI = Tucker-Lewis Index; RMSEA = Root Mean Square Error of Approximation with 95% confidence interval; AIC = Akaike Information Criterion; BIC = Bayesian Information Criterion; $\Delta\chi^2$ = chi-square difference test statistic; Δdf = degrees of freedom for difference test. Lower AIC and BIC values indicate better model fit accounting for parsimony.

Table S7. Pairwise zero-order correlations between all variables by timepoint and sex

Available as a separate Excel file: TableS7_ZeroOrderCorrelations.xlsx.

Table S8. Fit Indices for Full and Pruned Panel GVAR Models

Fit Index	Model (Full)	Model (Pruned)
CFI	0.89	0.89
TLI	0.86	0.88
RMSEA	0.026	0.024
RMSEA 90% CI	[0.025, 0.027]	[0.023, 0.024]
χ^2	5840.77	6076.43
df	770	925
AIC	665,598.37	665,524.03
BIC	667,495.52	666,307.32
EBIC	671,491.62	667,957.23
n	1,034	1,034
Parameters	264	109

Note. CFI = Comparative Fit Index; TLI = Tucker-Lewis Index; RMSEA = Root Mean Square Error of Approximation with 90% confidence interval; AIC = Akaike Information Criterion; BIC = Bayesian Information Criterion; EBIC = Extended Bayesian Information Criterion. n reflects the number of participants with complete data contributing to model estimation.

Bootstrapping procedures

To evaluate the stability of the estimated panel GVAR networks, we conducted 1,000 case-drop bootstrap iterations on the final pruned model. In each iteration, 25% of participants were randomly removed, and the full model was re-estimated using the same specification as in the primary analyses. The proportion of bootstrap samples in which each edge was retained is displayed in the supplementary correlation plots for the temporal and contemporaneous networks (Figure S5 and S6). Overall, within-domain edges (symptom–symptom and brain–brain associations) showed high stability across bootstrap samples, particularly in the contemporaneous network, where several core edges were retained in more than 75% of iterations. In contrast, cross-domain brain–symptom associations demonstrated more variable stability. In the temporal network, the edge from Depressed Mood to Global surface area was retained in approximately 32.5% of bootstrap samples, indicating low robustness. The temporal edge from Depressed Mood to mOFC thickness was retained in approximately 29.6% of iterations, and the temporal edge from Worthlessness to global surface area appeared in approximately 27.2% of iterations, both suggesting low stability and considerable sampling variability. In the contemporaneous network, cross-domain edges between Depressed Mood and surface area were retained in roughly 43.4% of bootstrap samples. In the between person network the cross-domain association between surface area and anhedonia appeared in 99.5% of iterations indicating high stability. Together, these results suggest that core within-domain structures are relatively stable, whereas cross-domain brain–symptom associations, particularly in the temporal network, should be interpreted with appropriate caution given their modest to unstable bootstrap consistency.

Figure S5. Case-Drop Bootstrap Stability – Temporal Network

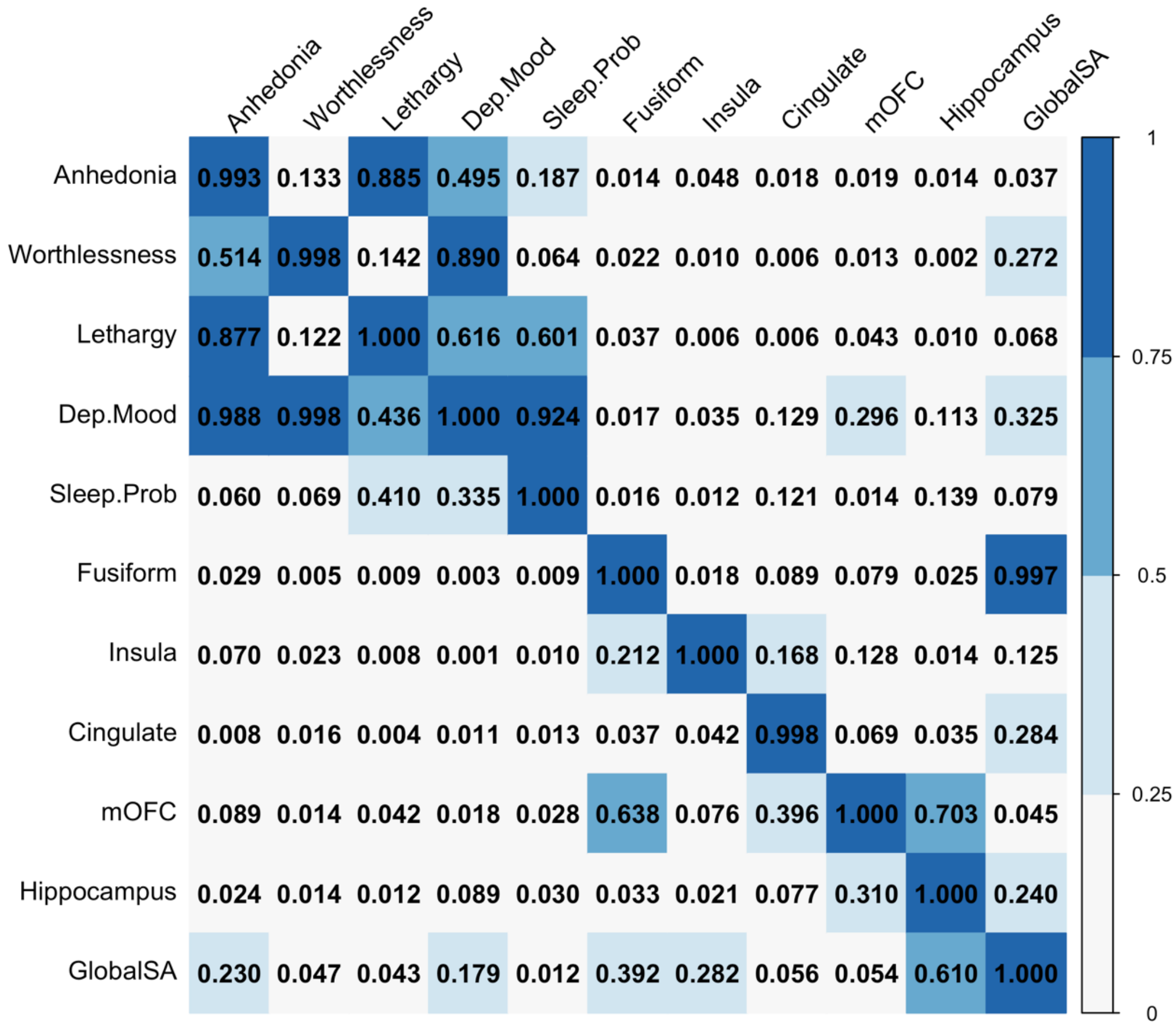

*Note***.** Proportion of bootstrap samples (1,000 case-drop iterations; 25% of participants removed per iteration) in which each temporal edge was retained in the pruned panel GVAR model. Values represent the proportion of times an edge was estimated as non-zero across bootstrap samples. Higher values indicate greater stability of the lagged within-person associations.

Figure S6. Case-Drop Bootstrap Stability – Contemporaneous Network

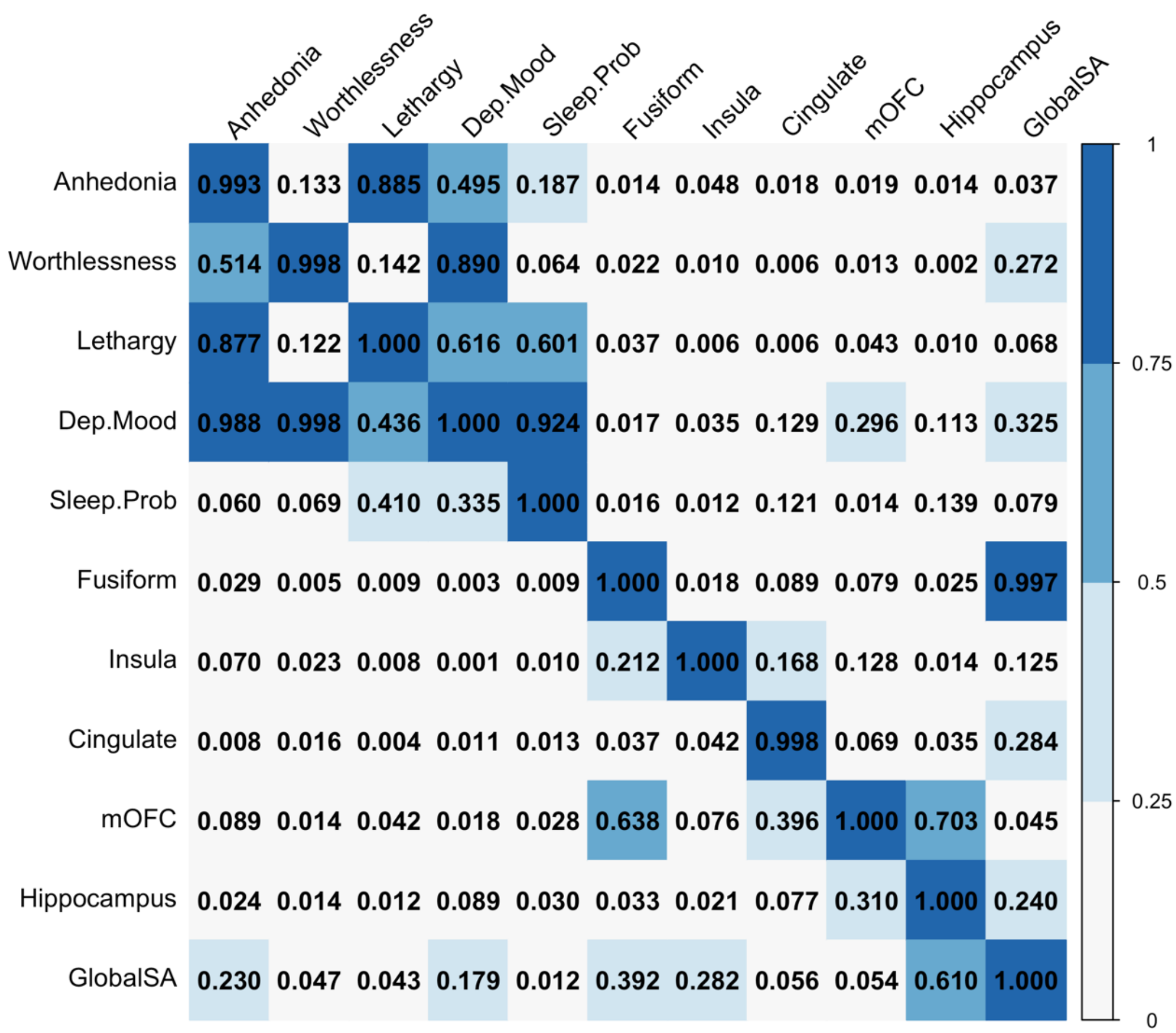

*Note***.** Proportion of bootstrap samples (1,000 case-drop iterations; 25% of participants removed per iteration) in which each contemporaneous edge was retained in the pruned panel GVAR model. Values reflect the consistency of within-person co-deviation associations across bootstrap samples. Higher proportions indicate greater edge stability.

Figure S7. Case-Drop Bootstrap Stability – Between Network

	Anhedonia	Worthlessness	Lethargy	Dep.Mood	Sleep.Prob	Fusiform	Insula	Cingulate	mOFC	Hippocampus	GlobalSA
Anhedonia	0.993	0.133	0.885	0.495	0.187	0.014	0.048	0.018	0.019	0.014	0.037
Worthlessness	0.514	0.998	0.142	0.890	0.064	0.022	0.010	0.006	0.013	0.002	0.272
Lethargy	0.877	0.122	1.000	0.616	0.601	0.037	0.006	0.006	0.043	0.010	0.068
Dep.Mood	0.988	0.998	0.436	1.000	0.924	0.017	0.035	0.129	0.296	0.113	0.325
Sleep.Prob	0.060	0.069	0.410	0.335	1.000	0.016	0.012	0.121	0.014	0.139	0.079
Fusiform	0.029	0.005	0.009	0.003	0.009	1.000	0.018	0.089	0.079	0.025	0.997
Insula	0.070	0.023	0.008	0.001	0.010	0.212	1.000	0.168	0.128	0.014	0.125
Cingulate	0.008	0.016	0.004	0.011	0.013	0.037	0.042	0.998	0.069	0.035	0.284
mOFC	0.089	0.014	0.042	0.018	0.028	0.638	0.076	0.396	1.000	0.703	0.045
Hippocampus	0.024	0.014	0.012	0.089	0.030	0.033	0.021	0.077	0.310	1.000	0.240
GlobalSA	0.230	0.047	0.043	0.179	0.012	0.392	0.282	0.056	0.054	0.610	1.000

Figure S8. Sex-specific between-person networks

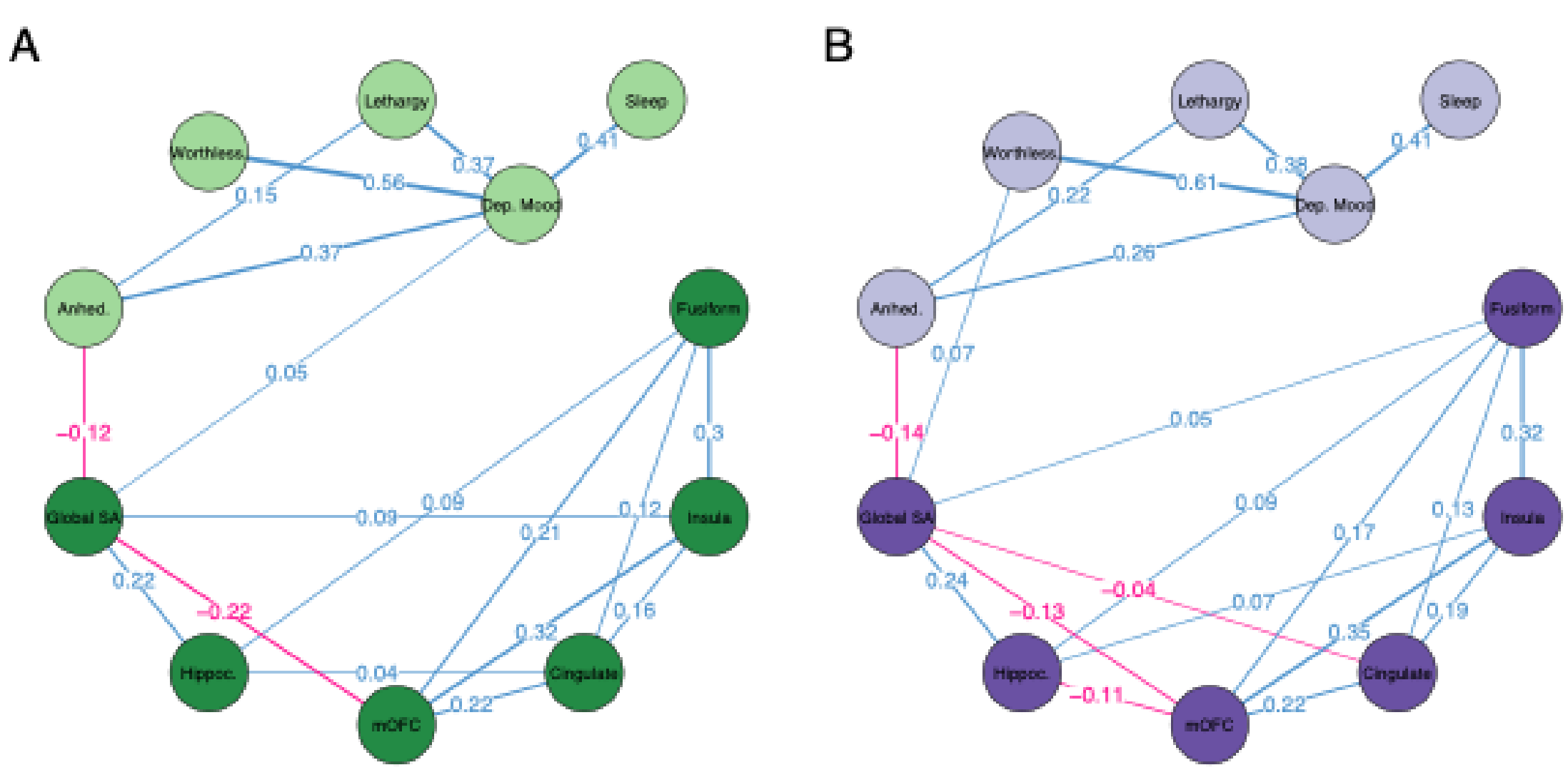

Note. Sex-specific between-person networks for males (A) and females (B). Male network (A): light green nodes represent depressive symptoms and dark green nodes represent structural brain metrics. Female network (B): light purple nodes represent depressive symptoms and dark purple nodes represent structural brain metrics. Blue edges indicate positive partial correlations and red edges indicate negative partial correlations. Edge transparency is uniform across all edges regardless of magnitude. No cross-domain associations were observed in either network.

Depression Sum Score

Composition and scoring

As a sensitivity analysis, we modelled the psychometrically validated CBCL DSM-5 Depressive Problems scale (DSM_Dep_Sum, 13 items) as a single aggregated node alongside the same six brain features. This scale was drawn directly from the ABCD tabulated data (mh_p_cbcl__dsm__dep_sum). A parallel analysis using a self-computed five-item sum score (Dep_Sum) aggregating only the symptom items from the main analysis yielded virtually identical results and is not reported separately.

Distribution

Descriptive statistics for the DSM depression sum score across timepoints are presented in Figure S9 alongside the individual item statistics in Figure S10. The distribution was positively skewed, consistent with the subclinical nature of the community sample, with the majority of participants scoring at or near zero at each timepoint.

Figure S9. Distribution of depression sum score across timepoints.

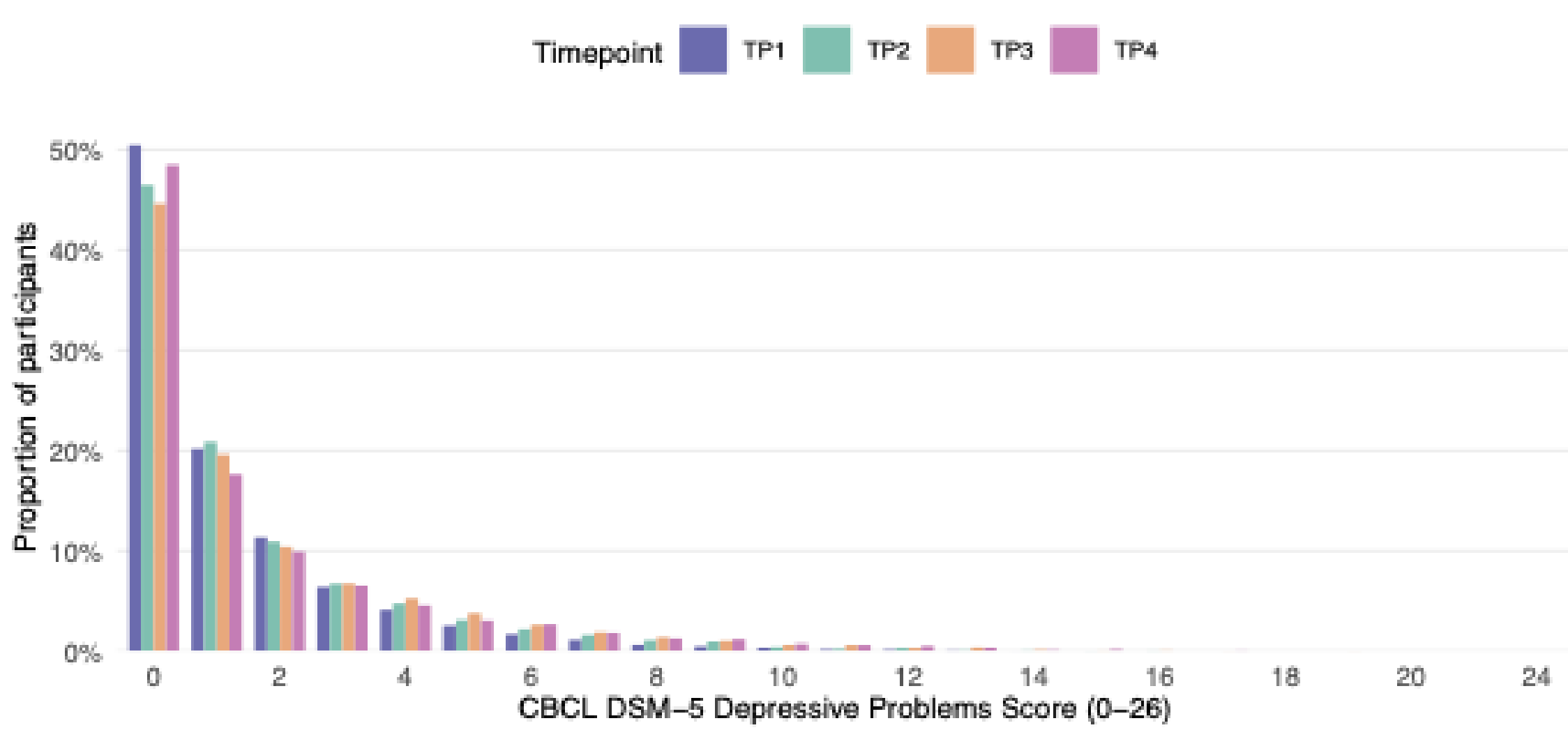

Note. Bars show the proportion of participants at each depression sum score value (0–26) separately for each timepoint. The sum score reflects the psychometrically validated CBCL DSM-5 Depressive Problems scale (DSM_Dep_Sum, 13 items), each rated on a 0–2 scale. The distribution is positively skewed at all timepoints, consistent with the subclinical nature of the community sample. Timepoint 1 represents baseline and timepoints 2–4 represent 2-year, 4-year, and 6-year follow-up assessments.

Figure S10. Distribution of CBCL depressive symptom item responses across timepoints, split by sex.

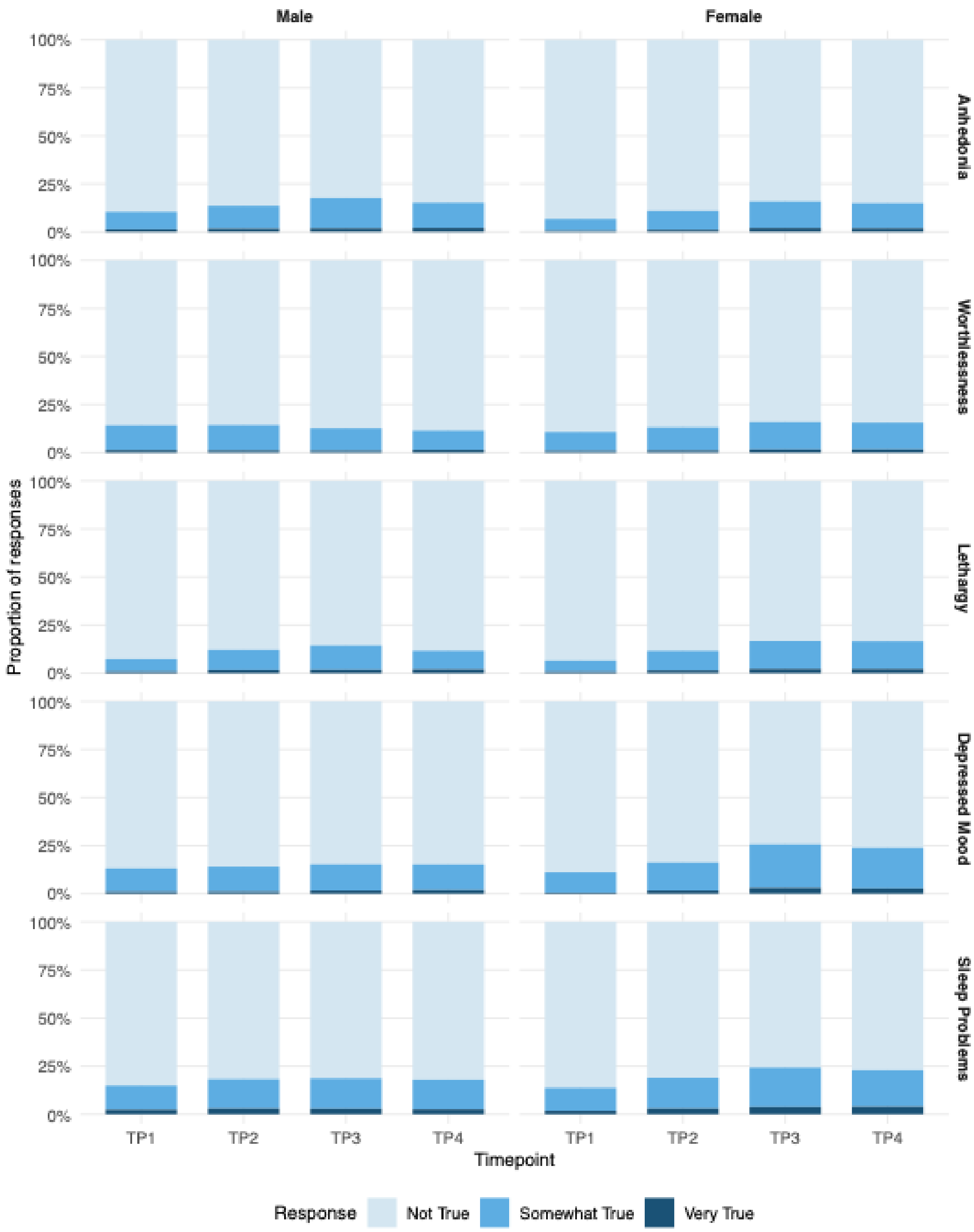

Note. Stacked bars show the proportion of responses in each category (Not True, Somewhat True, Very True) for each of the five depressive symptom items (anhedonia, worthlessness, lethargy, depressed mood, and sleep problems) across timepoints 1–4, separately for males and females. Items are drawn from the Child Behavior

Checklist (CBCL) parent report. Timepoint 1 represents baseline and timepoints 2–4 represent 2-year, 4-year, and 6-year follow-up assessments.

Relation to individual CBCL items and the five-item sum score

The DSM-5 Depressive Problems scale was strongly correlated with each of the five individual symptom items used in the main analysis (depressed mood: r = .769 [.765, .774]; anhedonia: r = .673 [.666, .679]; worthlessness: r = .673 [.666, .679]; lethargy: r = .648 [.642, .655]; sleep problems: r = .646 [.639, .653]; all p < .001), and with the self-computed five-item sum score (r = .920 [.919, .922], p < .001), indicating strong convergent validity.

Figure S11. Cumulative percentage of sample with MDD diagnosis across timepoints

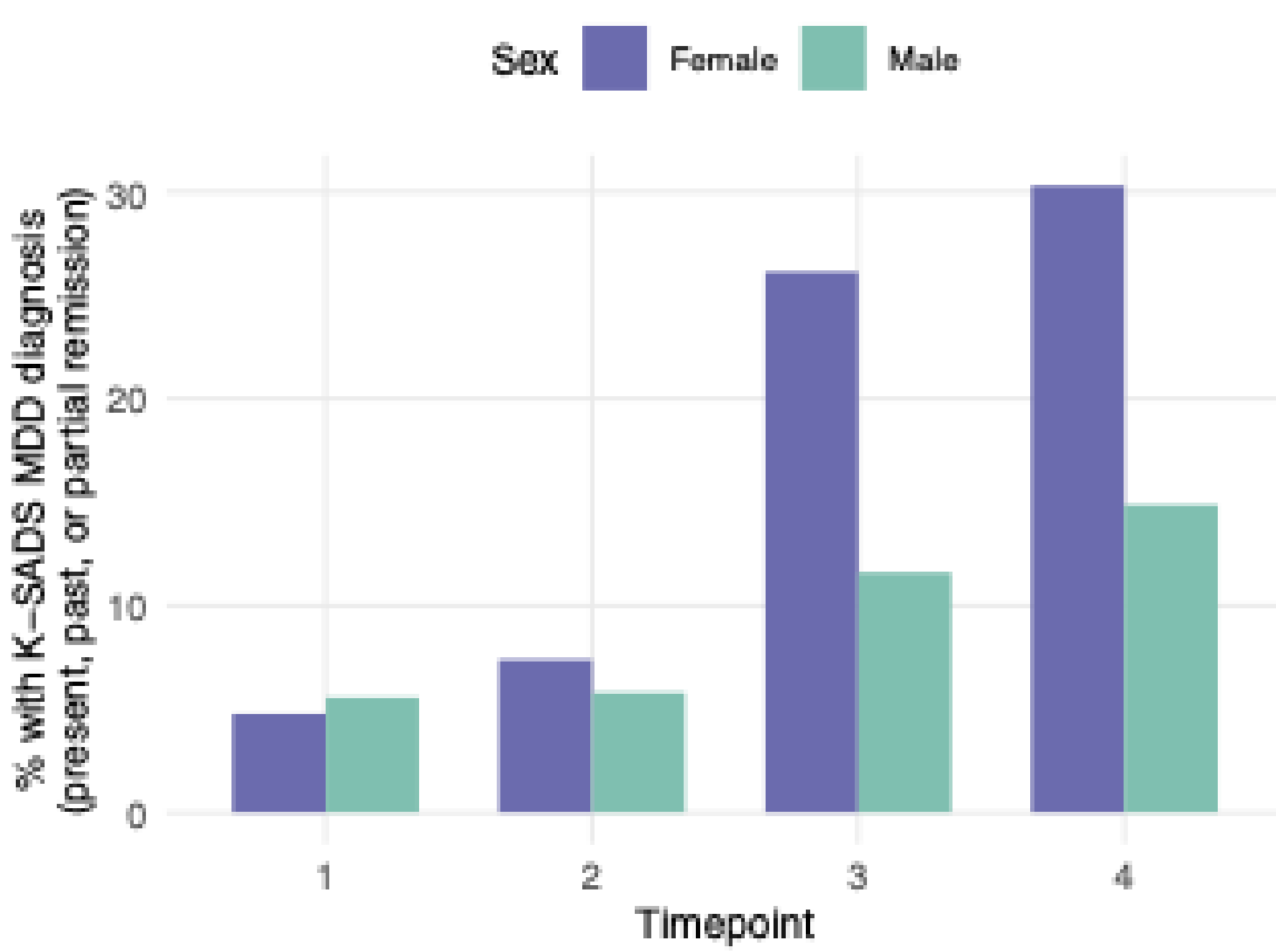

Figure S11. Prevalence of K-SADS Major Depressive Disorder diagnosis by timepoint and sex. Bars show the percentage of participants meeting criteria for MDD (present, past, or partial remission; parent or self-report) at each assessment wave, stratified by sex. Because the K-SADS "past" indicator reflects lifetime history up to the time of assessment, prevalence is cumulative across waves rather than reflecting new cases at each timepoint. When restricting to incident cases not previously identified at an earlier wave, new MDD positivity was 5.2%, 5.4%, 14.6%, and 13.8% at TP1 through TP4 respectively, with the marked increase at TP3-TP4 consistent with the well-documented rise in depression incidence during early-to-mid adolescence

Table S9. K-SADS Major Depressive Disorder diagnosis prevalence by timepoint

Timepoint	N	MDD present, parent (%)	MDD past, parent (%)	MDD partial remission, parent (%)	MDD present, self (%)	MDD past, self (%)	MDD partial remission, self (%)	MDD any (%)
TP1	9,560	0.21	2.60	0.19	0.48	2.09	0.20	5.20
TP2	6,569	0.29	3.19	0.45	0.47	2.99	0.37	6.54
TP3	5,202	0.89	1.71	5.11	2.32	3.88	9.23	18.30
TP4	6,250	1.01	2.65	6.48	2.34	4.60	10.34	22.17

Note. Present = current MDD diagnosis at time of assessment; Past = lifetime history of MDD prior to current assessment; Partial remission = MDD in partial remission at time of assessment. Parent and self (youth) report based on K-SADS-COMP diagnostic interview. MDD any = positive on any of the six indicators (present, past, or partial remission; parent or self-report).

Figure S12. Pruned panel GVAR networks in participants with a past or present MDD diagnosis (K-SADS). Temporal (A), contemporaneous (B), and between-person (C) networks

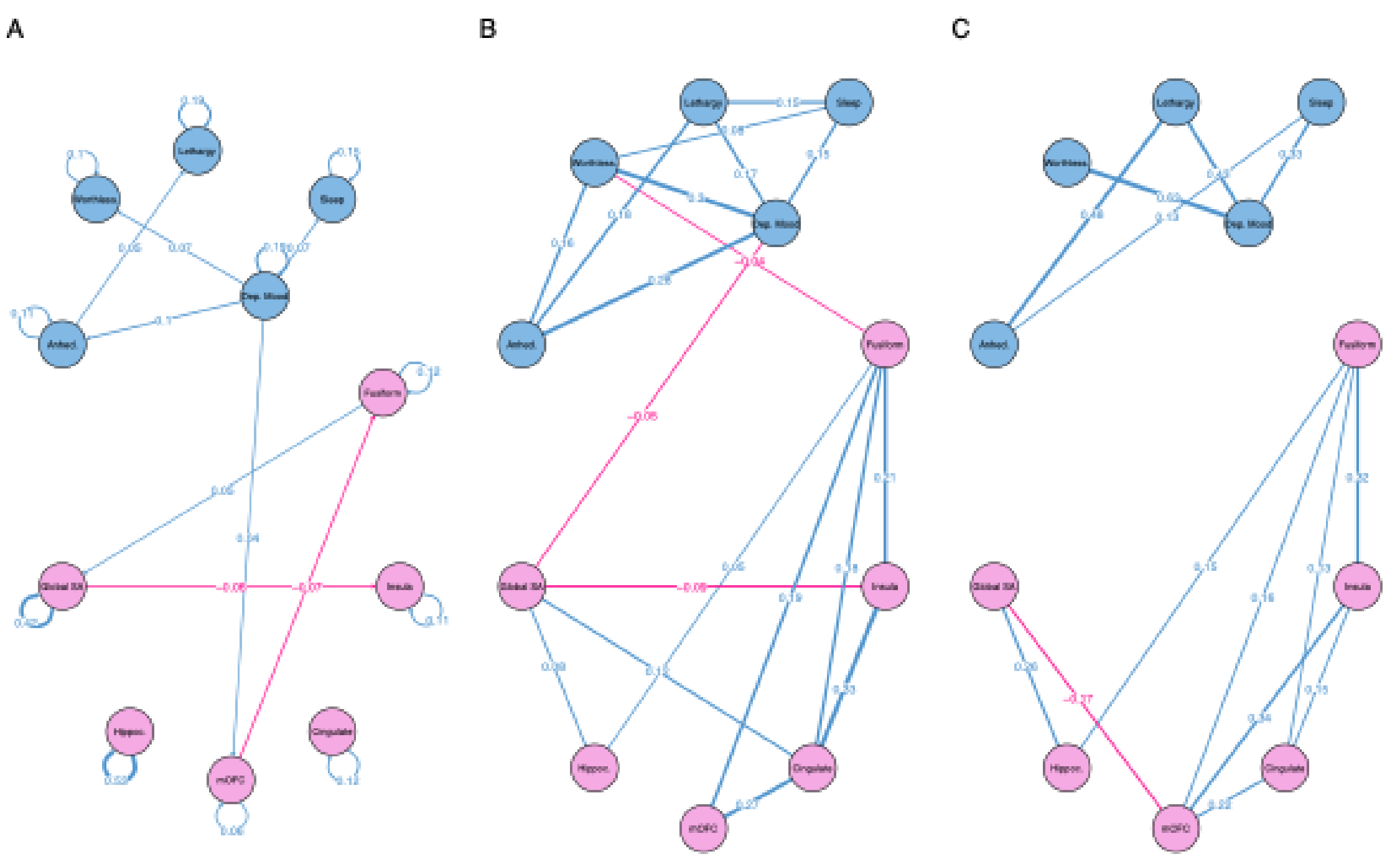

Note. Networks estimated in the subsample of participants screening positive for a Major Depressive Disorder diagnosis (present, past, or partial remission; parent or youth self-report) at one or more assessment waves (N = 2,450; 25.1% of the full analytic sample), based on K-SADS diagnostic interviews. Blue nodes represent depressive symptoms: anhedonia (Anhed.), worthlessness (Worthless.), lethargy, depressed mood (Dep. Mood), and sleep problems (Sleep). Pink nodes represent structural brain metrics: fusiform gyrus, insula, cingulate cortex, medial orbitofrontal cortex (mOFC), hippocampus (Hippoc.), and global surface area (Global SA). Blue edges indicate positive partial correlations and pink edges indicate negative partial correlations. Only edges surviving the pruning step (α = .05) are shown. Edge labels reflect partial correlation coefficients.

Table S10. Past-year psychotropic medication use by timepoint

Timepoint	N	Antidepressant non-SSRI (%)	Antidepressant SSRI (%)	Stimulant amphetamine (%)	Stimulant methylphenidate (%)
TP1	9,560	0.38	1.56	3.06	4.62
TP2	6,569	0.61	2.78	3.56	5.23
TP3	5,202	1.95	6.57	4.64	5.51
TP4	6,250	3.28	10.42	5.03	5.76

Note. Percentages reflect parent-reported past-year medication use categories from the ABCD Medication History inventory. Stimulant categories reflect ADHD medication use and are reported as a separate pharmacological confound independent of depression-related comorbidity.

Figure S13. Medication use across timepoint split by sex

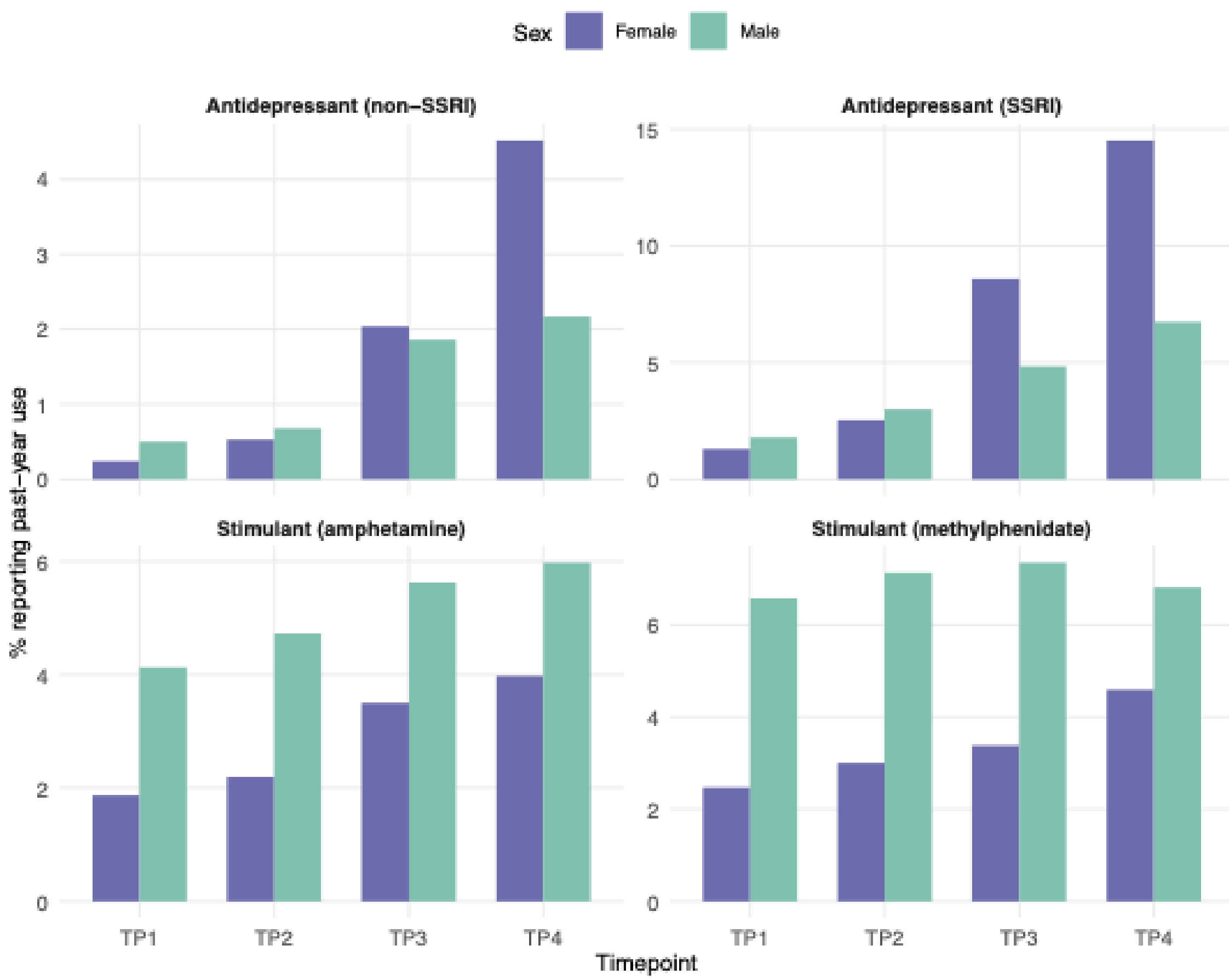

Note. Bar charts display the percentage of participants reporting past-year use of antidepressant and stimulant medications across four assessment timepoints, separately by sex, based on parent-reported medication history. Antidepressant use is reported separately for non-SSRI and SSRI classes; stimulant use is reported separately for amphetamine-based and methylphenidate-based medications. These data are provided as contextual characterization of psychotropic medication use in this community-based sample and were not used as covariates in the primary analyses. TP1 = baseline; TP2 = 2-year follow-up; TP3 = 4-year follow-up; TP4 = 6-year follow-up.

Figure S14. Externalizing and Internalizing symptom scores across timepoints by sex

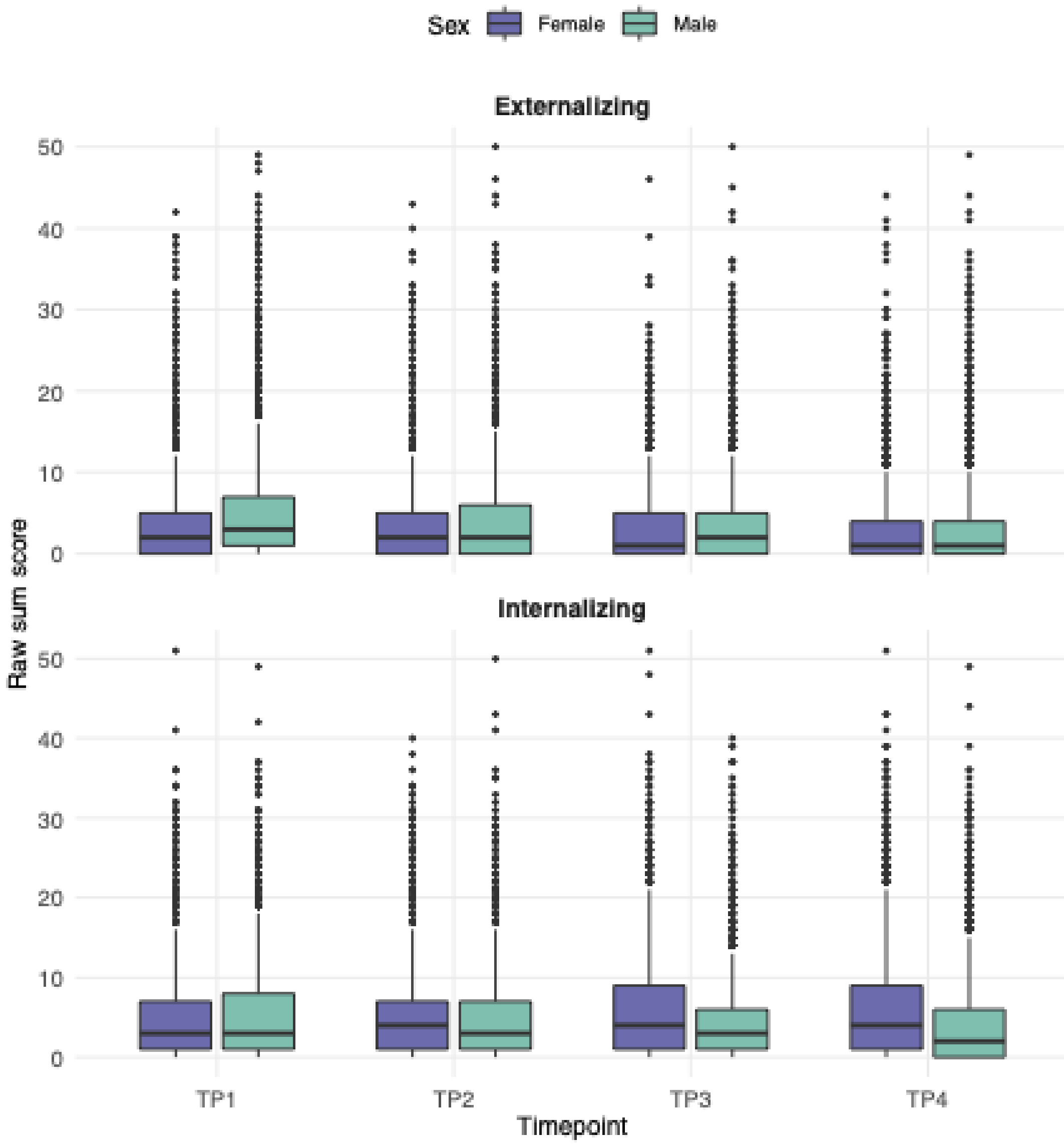

Note. Boxplots display the distribution of raw CBCL syndrome scale sum scores for internalizing and externalizing problems across four assessment timepoints, separately by sex. Boxes represent the interquartile range (IQR), with the horizontal line indicating the median. Whiskers extend to 1.5 × IQR; points beyond this range are displayed individually. Scores reflect parent-reported symptom burden in this community-based, non-clinical sample and are provided as contextual characterization of comorbid symptom levels. Higher scores indicate greater symptom severity. TP1 = baseline; TP2 = 2-year follow-up; TP3 = 4-year follow-up; TP4 = 6-year follow-up.

Table S11. CBCL Internalizing and Externalizing sum scores by timepoint and sex

Timepoint	Sex	N	Internalizing Mean	Internalizing SD	Externalizing Mean	Externalizing SD
TP1	Female	4,547	5.16	5.61	3.75	5.09
TP1	Male	5,013	5.21	5.63	5.16	6.46
TP2	Female	3,018	5.36	5.89	3.48	5.02
TP2	Male	3,551	4.84	5.51	4.48	5.90
TP3	Female	2,407	6.20	6.76	3.36	4.88
TP3	Male	2,795	4.63	5.64	4.03	5.83
TP4	Female	2,955	6.24	6.95	2.99	4.77
TP4	Male	3,295	4.21	5.59	3.43	5.41

Note. Internalizing and Externalizing scores reflect CBCL syndrome scale raw sum scores. Reported to characterize comorbid internalizing and externalizing symptom burden in this non-clinical, population-based sample.

Table S12. Saturated Edge weights

This table can be found in a separate excel file: TableS12_edge_weights_saturated_7.0

Pubertal Timing Sensitivity Analysis: Early vs. Late Developers Within Each Sex

In both females ($\Delta\chi^2 = 267.62$, df = 176, $p < .001$) and males ($\Delta\chi^2 = 235.44$, df = 176, $p = .002$), the χ^2 difference test indicated statistically significant heterogeneity in within-person network structure by pubertal timing. However, AIC favored the more parsimonious equal-constraints model in every comparison for both sexes (Tables S13–S14), in contrast to the sex-difference analysis where AIC and χ^2 converged on substantial within-person heterogeneity (ΔAIC = +150.93). This pattern suggests that pubertal timing differences, while statistically detectable given the large sample size, are smaller in practical magnitude than the sex differences identified in the primary analysis, and that the observed sex differences in within-person network structure are not simply attributable to differences in pubertal timing between groups. Between-person network structure did not differ significantly by pubertal timing in females ($\Delta\chi^2 = 49.09$, df = 55, $p = .699$), consistent with the primary finding of sex-invariant between-person associations. In males, a marginal between-person difference was

observed ($\Delta\chi^2 = 78.21$, df = 55, p = .022); however, AIC again favored the equal model, suggesting this difference is of limited practical importance.

Figure S15. Network plots split by sex and early vs. late pubertal timing

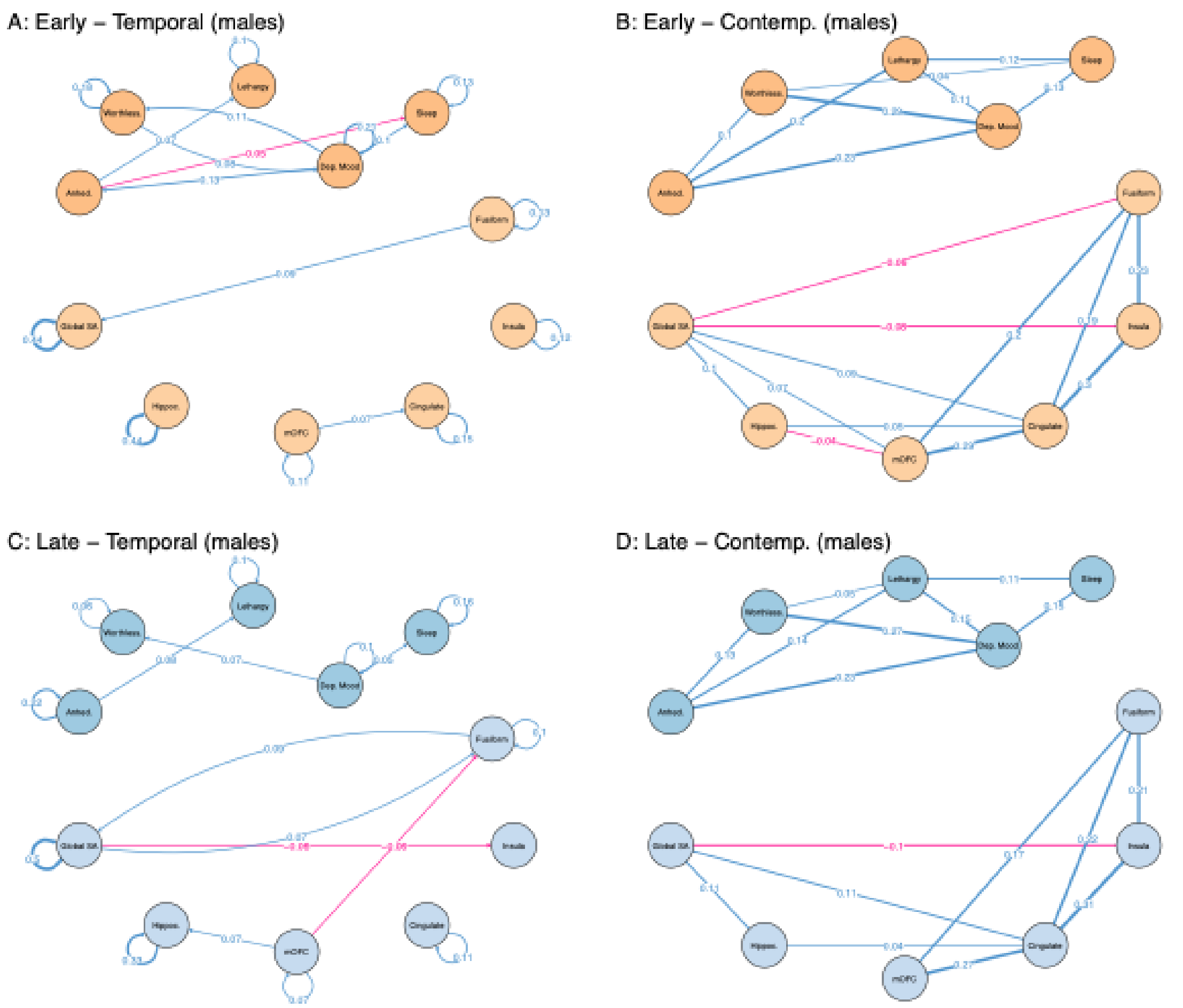

Note. Pruned temporal and contemporaneous networks estimated separately for early (orange nodes, panels A–B) and late (blue-grey nodes, panels C–D) pubertal timing groups within males. Pubertal timing was operationalized as the standardized residual of parent-reported Pubertal Development Scale (PDS) total score regressed on age within each sex, with early and late groups defined via within-sex median split. Blue edges indicate positive partial correlations and pink edges indicate negative partial correlations. Only edges surviving the pruning step ($\alpha = .05$) are shown. Edge labels reflect partial correlation coefficients. Between-person networks are not shown as they did not differ meaningfully by pubertal timing group (see Table S13).

Table S13. Pubertal Timing Invariance Testing — Females (Early vs. Late)

Comparison	Model	df	AIC	BIC	RMSEA	χ^2	$\Delta\chi^2$	Δdf	p
All networks	Different	1,540	283,419	286,766	.039	6,491.69	—	—	—
	Equal	1,771	283,273	285,156	.037	6,807.80	316.11	231	< .001
Within-person	Different	1,540	283,419	286,766	.039	6,491.69	—	—	—
	Equal (within)	1,716	283,335	285,566	.037	6,759.31	267.62	176	< .001
Between-person	Different	1,540	283,419	286,766	.039	6,491.69	—	—	—
	Equal (between)	1,595	283,358	286,357	.038	6,540.78	49.09	55	.699

Note. The "Different" model (configural) freely estimates all network parameters separately for early and late pubertal timing groups. The "Equal" model constrains all parameters to be equal across pubertal timing groups. "Equal (within)" constrains only within-person parameters; "Equal (between)" constrains only between-person parameters. AIC favoured the Equal model in all comparisons, indicating that pubertal timing differences, while statistically detectable, are small in practical magnitude. df = degrees of freedom; RMSEA = Root Mean Square Error of Approximation; AIC = Akaike Information Criterion; BIC = Bayesian Information Criterion; $\Delta\chi^2$ = chi-square difference statistic; Δdf = degrees of freedom for difference test.

Table S14. Pubertal Timing Invariance Testing — Males (Early vs. Late)

Comparison	Model	df	AIC	BIC	RMSEA	χ^2	$\Delta\chi^2$	Δdf	p
All networks	Different	1,540	321,261	324,660	.024	3,627.62	—	—	—
	Equal	1,771	321,111	323,022	.023	3,939.09	311.48	231	< .001
Within-person	Different	1,540	321,261	324,660	.024	3,627.62	—	—	—
	Equal (within)	1,716	321,145	323,410	.023	3,863.05	235.44	176	.002
Between-person	Different	1,540	321,261	324,660	.024	3,627.62	—	—	—
	Equal (between)	1,595	321,230	324,274	.024	3,705.83	78.21	55	.022

Note. The "Different" model (configural) freely estimates all network parameters separately for early and late pubertal timing groups. The "Equal" model constrains all parameters to be equal across pubertal timing groups. "Equal (within)" constrains only within-person parameters; "Equal (between)" constrains only between-person parameters. AIC favoured the Equal model in all comparisons, indicating that pubertal timing differences, while statistically detectable, are small in practical magnitude. df = degrees of freedom; RMSEA = Root Mean Square Error of Approximation; AIC = Akaike Information Criterion; BIC = Bayesian Information Criterion; $\Delta\chi^2$ = chi-square difference statistic; Δdf = degrees of freedom for difference test.

References

Beer, J. C., Tustison, N. J., Cook, P. A., Davatzikos, C., Sheline, Y. I., Shinohara, R. T., & Linn, K. A. (2020). Longitudinal ComBat: A method for harmonizing longitudinal multi-scanner imaging data. *NeuroImage*, *220*, 117129. https://doi.org/10.1016/j.neuroimage.2020.117129

Casey, B. J., Cannonier, T., Conley, M. I., Cohen, A. O., Barch, D. M., Heitzeg, M. M., Soules, M. E., Teslovich, T., Dellarco, D. V., Garavan, H., Orr, C. A., Wager, T. D., Banich, M. T., Speer, N. K., Sutherland, M. T., Riedel, M. C., Dick, A. S., Bjork, J. M., Thomas, K. M., … ABCD Imaging Acquisition Workgroup. (2018). The Adolescent Brain Cognitive Development (ABCD) study: Imaging acquisition across 21 sites. *Developmental Cognitive Neuroscience*, *32*, 43–54. https://doi.org/10.1016/j.dcn.2018.03.001

Desikan, R. S., Ségonne, F., Fischl, B., Quinn, B. T., Dickerson, B. C., Blacker, D., Buckner, R. L., Dale, A. M., Maguire, R. P., Hyman, B. T., Albert, M. S., & Killiany, R. J. (2006). An automated labeling system for subdividing the human cerebral cortex on MRI scans into gyral based regions of interest. *NeuroImage*, *31*(3), 968–980. https://doi.org/10.1016/j.neuroimage.2006.01.021

Epskamp, S. (2025). *psychonetrics: Structural equation modeling and confirmatory network analysis* [Manual]. https://CRAN.R-project.org/package=psychonetrics

Fischl, B., & Dale, A. M. (2000). Measuring the thickness of the human cerebral cortex from magnetic resonance images. *Proceedings of the National Academy of Sciences*, *97*(20), 11050–11055. https://doi.org/10.1073/pnas.200033797

Fischl, B., Salat, D. H., Busa, E., Albert, M., Dieterich, M., Haselgrove, C., van der Kouwe, A., Killiany, R., Kennedy, D., Klaveness, S., Montillo, A., Makris, N., Rosen, B., & Dale, A. M. (2002). Whole brain segmentation: Automated labeling of

neuroanatomical structures in the human brain. *Neuron*, *33*(3), 341–355. https://doi.org/10.1016/s0896-6273(02)00569-x

Fortin, J.-P., Cullen, N., Sheline, Y. I., Taylor, W. D., Aselcioglu, I., Cook, P. A., Adams, P., Cooper, C., Fava, M., McGrath, P. J., McInnis, M., Phillips, M. L., Trivedi, M. H., Weissman, M. M., & Shinohara, R. T. (2018). Harmonization of cortical thickness measurements across scanners and sites. *NeuroImage*, *167*, 104–120. https://doi.org/10.1016/j.neuroimage.2017.11.024

Hagler, D. J., Hatton, SeanN., Cornejo, M. D., Makowski, C., Fair, D. A., Dick, A. S., Sutherland, M. T., Casey, B. J., Barch, D. M., Harms, M. P., Watts, R., Bjork, J. M., Garavan, H. P., Hilmer, L., Pung, C. J., Sicat, C. S., Kuperman, J., Bartsch, H., Xue, F., … Dale, A. M. (2019). Image processing and analysis methods for the Adolescent Brain Cognitive Development Study. *NeuroImage*, *202*, 116091. https://doi.org/10.1016/j.neuroimage.2019.116091

Hoekstra, R. H. A., Epskamp, S., Nierenberg, A. A., Borsboom, D., & McNally, R. J. (2024). Testing similarity in longitudinal networks: The Individual Network Invariance Test. *Psychological Methods*. https://doi.org/10.1037/met0000638

Norbom, L. B., Ferschmann, L., Parker, N., Agartz, I., Andreassen, O. A., Paus, T., Westlye, L. T., & Tamnes, C. K. (2021). New insights into the dynamic development of the cerebral cortex in childhood and adolescence: Integrating macro- and microstructural MRI findings. *Progress in Neurobiology*, *204*, 102109. https://doi.org/10.1016/j.pneurobio.2021.102109
